# Pandemics past, present, and future: progress and persistent risks

**DOI:** 10.1172/JCI179519

**Published:** 2024-04-01

**Authors:** Arturo Casadevall

**Affiliations:** Department of Molecular Microbiology and Immunology, Johns Hopkins School of Public Health, Baltimore, Maryland, USA.

When the *JCI* began publishing in 1924, the world was recovering from the end of the First World War and the lingering aftermath of the 1918 influenza pandemic. The 100th anniversary of the *JCI* occurs nearly five years since the initial appearance of COVID-19, caused by SARS-CoV-2. I was a *JCI* deputy editor during the early years of the COVID-19 pandemic, when I witnessed firsthand how the pandemic affected journal operations and how the *Journal* published a series of important articles that defined the disease and the immune response and helped catalyze early medical responses (1). The *JCI* has now published through five pandemics, three caused by influenza virus in 1957, 1968, and 2009, one caused by the retrovirus HIV that began in 1981, and another caused by the coronavirus SARS-CoV-2 in 2019. In this essay, I will reflect on what we have learned from these five pandemics and try to identify themes that could help with future global infectious disease outbreaks.

## Pandemics begin suddenly and never really end

All pandemics surprise humanity. Whereas the beginning of a pandemic can usually be established, it is not clear when pandemics end, or whether they really end. The black death of the Middle Ages caused by *Yersinia pestis* began in 1347, abated in 1351, recurred several times, and may have left the bacterium endemic in affected areas (2). For the five pandemics that occurred since the launching of the *JCI*, none has really ended. At the time of this writing, virus descendants from the influenza 2009 strain, HIV, and SARS-CoV-2 pandemics are still with us. In fact, all seasonal and pandemic influenza virus strains for the past century have some ancestry to the 1918 virus, which has prompted Taubenberger and Morens to state that “the impact of the pandemic virus — now known to be an H1N1 influenza A virus — was not, however, limited to 1918–1919. The 1918 influenza A virus became a ‘founder virus,’ initiating a ‘pandemic era’ by evolving into progeny pandemic viruses through a number of separate genetic reassortment events” (3). Each pandemic has in common the introduction into the human population of a new communicable agent for which there was initially little to no immunity that then spread widely and became established, thus changing the future dynamics of the host-microbe interaction.

## Pandemic agents are unpredictable

In 1979, I learned in medical school that retroviruses were good systems to study how viruses caused cancer but were not agents of human disease and that coronaviruses were minor pathogens that caused annoying upper respiratory infections. I relate this anecdote because two of the subsequent pandemics — those involving HIV and SARS-CoV-2 — were caused by a retrovirus and coronavirus, respectively, highlighting how medical knowledge evolves. In 1980, the human T cell leukemia virus was discovered and subsequently shown to cause T cell lymphoma, establishing that retroviruses were pathogenic for humans and foreshadowing the HIV pandemic, which would be caused by another retrovirus (4). Similarly, the medical world was surprised in 2003 by the outbreak of a respiratory coronavirus known as SARS-CoV, which caused pneumonia with high mortality. Fortunately, SARS-CoV did not develop into a pandemic. In 2012, another coronavirus (MERS-CoV) was described in the Middle East that caused severe respiratory disease with high mortality but was less contagious, and the outbreak has remained regional. The near brushes with SARS-CoV and MERS-CoV were not sufficient to illustrate the pandemic danger posed by coronaviruses, and humanity was surprised by the COVID-19 pandemic. Prior to COVID-19, much of the concern in medical circles was the possibility of a new influenza pandemic caused by a highly pathogenic avian influenza virus, such as H5N1, illustrating a weakness in the preparedness mindset that prioritized agents known to cause prior pandemics. One common denominator for the major pandemics since 1918 is a likely zoonotic origin for the viruses causing the 1918 influenza (3), HIV (5), 2019 H1N1 (6), and COVID-19 (7) pandemics and a probable zoonotic genetic input for the 1956 (H2N2) and 1968 (H3N2) influenza viruses (8). Hence, while pandemics are unpredictable, contact with the animal world can be anticipated to be a continuing source of new pandemic agents.

## Response to pandemics shows the progress of science

The five pandemics in the time of the *JCI*, together with the 1918 influenza pandemic, show how the progress of science over the past century has mitigated the catastrophic potential of infectious disease outbreaks (Table 1). For the 1918 influenza pandemic, the etiologic agent was unknown, as the isolation of the causative agent would have to wait until 1933, when the influenza virus was first isolated from humans. For the 1918 influenza pandemic, there were no vaccines, antiviral drugs, or supplemental oxygen, and the only specific therapy was convalescent serum, which retrospective analysis suggested reduced mortality by approximately 20% (9). The first pandemic during the *JCI*’s existence, and the second for the 20th century, was the 1957 to 1958 influenza pandemic, which began in Asia with the emergence of the H2N2 influenza strain. Three articles published in the *JCI* in 1959 illustrate the tremendous progress in medical knowledge that had occurred in the four decades since the 1918 influenza pandemic, including the discovery of the virus and methods for its cultivation, the development of vaccines, and the emergence of clinical immunology as a field. The first article reported less severe disease among the cohort that had been previously vaccinated, anticipating what is now accepted that vaccination reduces disease severity and death even when it does not prevent disease (10). The second article reported the occurrence of bacterial pneumonia as a major late complication of influenza pneumonia and noted that many cases were caused by *Staphylococcus aureus* (11), but by 1957 antibiotic therapy was available, reducing mortality. The third article detailed the isolation of the influenza virus from throat washing and lung tissue of patients who died, compared various methods of virus culturing, and included an electron micrograph of virions from culture, demonstrating tremendous progress in virological techniques (12).

In contrast to the first three pandemics of the 20th century, each caused by the influenza virus, the HIV pandemic involved a retrovirus not previously known to the medical field. By the early 1980s, advances in molecular biology allowed the identification of the virus three years after the first cases were identified, and antiviral therapy was available later in the decade, such that the resulting disease, acquired immunodeficiency syndrome (AIDS), went from being uniformly lethal to a manageable disease, if treated properly. In comparison with the four previous pandemics, the COVID-19 pandemic stands alone in the rapid development of antibody therapies, antiviral drugs, and effective vaccines within a year of the beginning of the outbreak. This rapidity illustrates the progress of science, which included the development of mRNA vaccines that proved remarkably effective in preventing death, although the rapid evolution of SARS-CoV-2 meant frequent breakthrough infections among the vaccinated. Administration of mRNA vaccines during pregnancy produced a robust antibody response that transferred immunity to the fetus within 15 days of vaccination (13). This result encouraged vaccination during pregnancy as a means of protecting newborns against SARS-CoV-2. Although the application of mRNA technology to vaccine development for COVID-19 was novel, this technology relied on decades of immunological, virological, and biochemical research and showed how earlier societal investments in basic research paid off during that pandemic (14). For the pandemics of 1968 and 2009, vaccines were available, but for HIV, no successful vaccine has yet been developed despite considerable research efforts.

## New infectious disease assaults from nature are inevitable

The first 23 years of the 21st century saw at least eight major viral outbreaks, including West Nile, SARS-CoV, H1N1, MERS-CoV, Zika, Ebola, SARS-CoV2, and mpox, three of which were new viruses and two of which resulted in pandemics. Given this record, we can expect continuing infectious disease assaults from nature in the years ahead. Whereas viruses loom large among potential threats, the black death, which is arguably the greatest calamity in recorded times, was caused by the bacterium *Yersinia pestis*. Humanity tends to worry about known threats and, consequently, is frequently surprised. In military history, there is the well-known saying that generals always prepare to fight the last war, but medicine should not fall into this trap, given that two of the last five pandemics were caused by new agents. In this regard, I note that fungal diseases are not high in threat assessments because there is no prior experience with pandemics caused by fungi. However, with regard to fungi, nature provides clear warnings such as, for example, the ongoing global pandemic of chytridiomycosis that has led to the extinction of multiple frog species and the fungi that are currently decimating numerous ecosystems that include bats in North America and salamanders in Europe (15). Although it is not possible to predict the cause of the next pandemic, preparedness must include a holistic assessment of possible threats, including agents that have not been historically implicated in human disease.

## Human nature as an ally to pandemic microbes

Despite the tremendous medical advances that spanned the century between the 1918 influenza and COVID-19 pandemics, human nature was constant and often acted as an ally to the viruses. In 1918, despite no knowledge about the causative organism, public health authorities correctly surmised that they were dealing with a respiratory pathogen and passed mask mandates that were resisted by many citizens. A century later, COVID-19 was known to be a respiratory pathogen, and masks were again recommended by public health authorities and resisted by many citizens. Both the 1918 influenza and COVID-19 pandemics saw citizen resistance to public health mandates, although in the case of COVID-19, some of the resistance was political (16). Even the development of safe and effective vaccines found resistance, and in the United States alone, vaccine hesitancy cost over 200,000 lives (17). However, the problem of faulty interpretation of medical evidence was not limited to the general public, given the fact that the medical profession misinterpreted the clinical evidence available for hydroxychloroquine, corticosteroids, and convalescent plasma, leading to actions that were associated with higher mortality (18). Hence, future progress in pandemic responses must address the social and behavioral issues that can conspire to interfere with effective measures, and that includes improved training for physicians in the evaluation of evidence.

## Climate change and pandemics

The world is currently experiencing unprecedented rapid global warming because of anthropogenic gas emissions. Temperature affects every chemical reaction necessary for life, and rapid global warming is an existential threat for many species, including our own. Much has been written about the effects of climate change on infectious diseases, and a consensus is emerging that it can amplify the progression of infectious disease outbreaks to pandemics (19). The confluence of climate change with a rising human population and an increasingly degraded environment create conditions propitious for new pandemics. In this regard, global warming will force changes to human and animal behavior that can significantly increase the likelihood of zoonotic spillovers and trigger new pandemics (20). In addition, global warming will force the adaptation of many microbes with human pathogenic potential to survive at higher temperatures, such that these microbes can then thrive at human temperatures and defeat our thermal defenses. The emergence of *Candida auris*, a fungal pathogen that has now spread globally after simultaneous, independent emergences, has been proposed to be a result of climate change (21) and may be a harbinger of new future infectious disease threats, some of which could have pandemic potential.

## Seven evolving themes from a century of pandemics

Looking back through the five pandemics in the century of the *JCI*’s existence allows one to discern seven themes that are likely to recur as humanity faces new dangers from infectious diseases in the years ahead. (a) Pandemics are unpredictable. (b) Most pandemics never end, as the agent evolves to find a continued niche in human populations. (c) Continued investment in science and preparedness is the best insurance policy against future pandemics. (d) Pandemics with new infectious disease agents pose the greatest danger, since countermeasures such as vaccines and new drugs will not be immediately available to contain the outbreak. (e) Human nature is often an ally of infectious disease outbreaks and spread, with human actions potentially interfering with public health efforts to mitigate suffering and death. (f) Progress against future pandemics requires not only scientific advances but also improvements in how physicians evaluate evidence as well as advances in the social sciences and communication to better understand human behavior and social structures in the response to emergencies. (g) Climate change due to anthropogenic warming heightens the risk of new pandemics by changing such variables as the introduction of new infectious diseases and a lessening of societal resilience in responding to heat-related stresses on agriculture, economics, and infrastructure.

Each pandemic brought untold suffering to those affected, but each pandemic was also accompanied by great learning and much scientific progress. We are just beginning to process the lessons from the COVID-19 pandemic, and I am optimistic that they will also catalyze great progress. Each pandemic left us in better shape to fight future pandemics because humanity learned and made investments in science and public health that increased preparedness against the unpredictable. There are more pandemics ahead, and such investments must continue to mitigate the great danger they pose to humanity.

## Figures and Tables

**Table 1:**
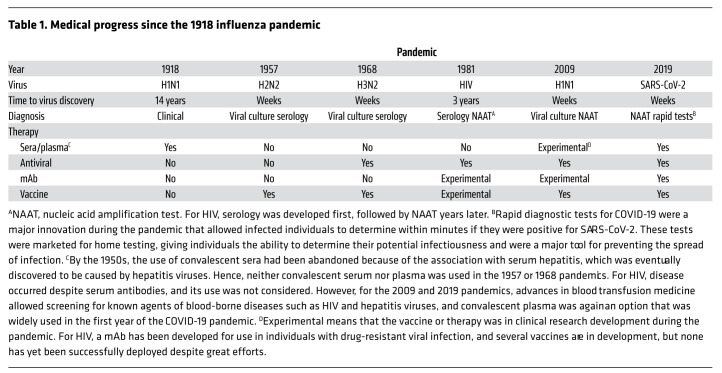
Medical progress since the 1918 influenza pandemic

## Version 1 04/01/2024

Electronic publication
